# Modes and mechanisms for the inheritance of mitochondria and plastids in pathogenic protists

**DOI:** 10.1371/journal.ppat.1012835

**Published:** 2025-01-23

**Authors:** Sophie L. Collier, Sarah N. Farrell, Christopher D. Goodman, Geoffrey I. McFadden

**Affiliations:** School of BioSciences, The University of Melbourne, Parkville, Victoria, Australia; Joan and Sanford I Weill Medical College of Cornell University, UNITED STATES OF AMERICA

## Abstract

Pathogenic protists are responsible for many diseases that significantly impact human and animal health across the globe. Almost all protists possess mitochondria or mitochondrion-related organelles, and many contain plastids. These endosymbiotic organelles are crucial to survival and provide well-validated and widely utilised drug targets in parasitic protists such as *Plasmodium* and *Toxoplasma*. However, mutations within the organellar genomes of mitochondria and plastids can lead to drug resistance. Such mutations ultimately challenge our ability to control and eradicate the diseases caused by these pathogenic protists. Therefore, it is important to understand how organellar genomes, and the resistance mutations encoded within them, are inherited during protist sexual reproduction and how this may impact the spread of drug resistance and future therapeutic approaches to target these organelles. In this review, we detail what is known about mitochondrial and plastid inheritance during sexual reproduction across different pathogenic protists, often turning to their better studied, nonpathogenic relatives for insight.

## Protists are important—And so are their endosymbiotic organelles

Protists are abundant, diverse, predominantly unicellular eukaryotes. They are ubiquitous, and many are pathogenic to humans, animals, plants, and even other protists. Almost all protists contain a mitochondrion, or derived version thereof, and many contain a plastid. Mitochondria and plastids arose from endosymbiotic bacteria and retain a relic of the bacterial genome of their predecessors. In parasitic protists, these organelles provide essential metabolic pathways that can make them important drug targets. Consequently, mutations in the organelle genome can confer drug resistance. It is, therefore, important that we understand the inheritance of mitochondria and plastids in these microorganisms. In this review, we explore what is known about mitochondrial and plastid inheritance during sexual reproduction across pathogenic protists, in many instances turning to their better studied nonpathogenic relatives or other more distant, but better-characterised eukaryotes for insight.

**Fig 1:**
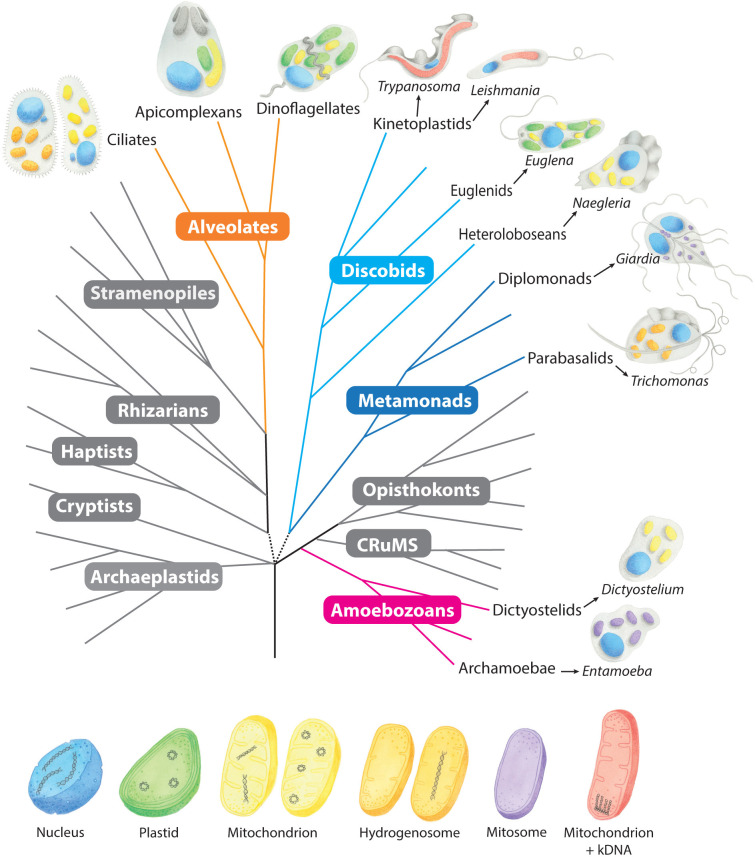
A phylogenetic tree of eukaryotes showing the variety of endosymbiotic organelles in pathogenic and nonpathogenic protists. The main lineages are redrawn according to Keeling and Burki (2019) and Husnik and colleagues (2021) and are depicted according to current consensus phylogeny [74,75]. Protists and organelles are hand-painted by Sarah N. Farrell. Clades and supergroups of interest are highlighted in colour. Each protist contains a nucleus (or a micronucleus and macronucleus in Ciliata) and endosymbiotic organelles. Organelles are colour coded according to the legend at the bottom of the figure. Classical mitochondria (yellow) can contain either linear or circular organellar genomes (as specified for each protist in Table 1 and the body text). Hydrogenosomes (orange) may also contain a linear genome or lack a mitochondrial genome entirely. The mitochondrion of *Trypanosoma* and *Leishmania* contains kinetoplast DNA (kDNA) comprised of DNA minicircles and maxicircles (red).

Except for *Monocercomonoides*, all protists possess mitochondria, or reduced forms of mitochondria known as mitochondrion-related organelles (MROs) [1]. These structures are a feature of both mutualistic and parasitic protists belonging to the Alveolata clade and protist supergroups including Euglenozoa, Metamonada, Amoebozoa, and Percolozoa (Fig 1 and Table 1). It is widely held that the mitochondrion was acquired through primary endosymbiosis of an α-proteobacterium that was internalised by another cell 1.5 to 2 billion years ago [2,3]. The resulting chimeric cell was ancestral to all modern-day eukaryotes. A hallmark of mitochondrial endosymbiosis is the presence of 2 membranes surrounding every mitochondrion.

Many protists contain a second organelle of endosymbiotic origin known as a plastid (Table 1). Plastids originated through endosymbiotic internalisation of a cyanobacterium by a eukaryotic phagotroph approximately 1.2 billion years ago [4–6]. Over time, this ancestral, photosynthetic eukaryote diverged into glaucophytes (none of which are pathogenic), green algae and land plants (various of which are parasitic, even to humans), and red algae (a few of which are parasitic on other red algae). At some undefined point, an early ancestor of Apicomplexa, and likely also the sister group of dinoflagellates, engulfed a eukaryotic red alga which became an established plastid within the host and eventually evolved into the non-photosynthetic apicoplast within parasites like *Plasmodium* and *Toxoplasma* [7] (Fig 1). The presence of 4 membranes surrounding the apicoplast is a signature of this secondary endosymbiosis [8]. The inner 2 membranes are the cyanobacterial (gram negative) derived membranes of the red algal plastid, the third membrane is hypothesised to be derived from the plasma membrane of the engulfed red alga, and the outer membrane is a remnant of the host phagosome that enclosed the endosymbiont during initial endocytosis [4].

**Table 1:** Summary of endosymbiotic organelles and organellar genome contents of the protists discussed in this review.

Protist group	Species	Endosymbiotic organelles	Organelle genome size (kb)	Organelle genome features	Source
**Dinoflagellates**	Various	Mitochondria	6–10 fragments (up to ~326 total in *Symbiodinium minutum*)	Linear, highly fragmented	[Reviewed in 9–11]
		Plastids	1.8–6 kb per minicircle&	Numerous minicircles^&^	[Reviewed in 12–15]
**Apicomplexans**	*Plasmodium*	Mitochondrion	6.0	Linear, single fragment	[16,17]
		Apicoplast	29.6–34.2*	Circular, single fragment	[16,18,19]
	*Toxoplasma*	Mitochondrion	5.9	Linear, fragmented	[20,21]
		Apicoplast	35.0	Circular, single fragment	[19,22]
	*Eimeria*	Mitochondrion	6.2	Linear, single fragment	[23,24]
		Apicoplast	34.8	Circular, single fragment	[19,25]
	*Babesia*	Mitochondria	6.2–11.1*	Linear, single fragment	[26–30]
		Apicoplast	28.7–35.1*	Circular, single fragment	[19,26,31–34]
	*Theileria*	Mitochondria	6.6–8.2*	Linear, single fragment	[30,35–38]
		Apicoplast	31.7–47.8*	Circular, single fragment	[19,37]
	*Cryptosporidium*	Mitosome	None	N/A	[Reviewed in 39]
	Gregarines	Mitosome	None	N/A	[40]
**Ciliates**	*Nyctotherus ovalis*	Hydrogenosomes	~41.6 (incomplete sequence)	Hypothesised to be linear (not directly studied)	[41–43]
	*Paramecium*	Mitochondria	40.0–44.0*	Linear, single fragment	[44,45]
**Percolozoans**	*Naegleria*	Mitochondria	49.5	Circular, single fragment	[46]
**Euglenozoans**	*Euglena*	Mitochondria	~1.0–9.0 (incomplete sequence)	Linear, fragmented	[47–49]
		Plastids	143.2	Circular, single fragment	[48,50]
	*Leishmania*	Mitochondrion	20–40 (maxicircles) ~1–2.5 (minicircles)	Comprised of 1,000 s of mini and 25–50 maxicircles	[51–53]
	*Trypanosoma*	Mitochondrion	20–40 (maxicircles) ~1–2.5 (minicircles)	Comprised of 1,000 s of mini and 25–50 maxicircles	[52–54]
**Metamonads**	*Giardia*	Mitosomes	None	N/A	[55]
	*Trichomonas*	Hydrogenosomes	None	N/A	[56]
**Amoebozoans**	*Dictyostelium*	Mitochondria	55.6	Circular, single fragment	[57]
	*Entamoeba*	Mitosomes	None	N/A	[58]

*Species dependent.

^&^In peridinin plastids specifically, plastid genomes vary in other “replacement plastid” species.

Dinoflagellates are close relatives of Apicomplexa that have adopted numerous lifestyles including parasitism. Some free-living dinoflagellates are also pathogenic to humans via the toxins they produce. Many dinoflagellates possess plastids of red algal origin also acquired through a secondary endosymbiosis event. These plastids differ from apicoplasts in that they are surrounded by 3 membranes. Many are photosynthetic, containing chlorophylls *a* and *c*, and the light-harvesting carotenoid pigment peridinin [59–61]. Peridinin plastids, and plastids in the related chromerid algae, likely share a common endosymbiotic origin with apicoplasts of Apicomplexa [62–65] (Fig 1). Somewhat surprisingly, many dinoflagellates have undergone further endosymbiotic events to replace their original peridinin plastid with new photosynthetic endosymbionts derived from a wide range of microorganisms including green algae, cryptophytes, haptophytes, or diatoms [66–68]. Such replacements can be secondary, tertiary, or even higher order endosymbioses [69]. Other dinoflagellates retain plastids that lack photosynthesis altogether, instead leading heterotrophic or parasitic lifestyles. At least one of these species, *Haematodinium*, has lost the plastid altogether [70,71].

Euglenids such as *Euglena*, procured plastids by secondary endosymbiosis independently of the alveolate dinoflagellates and apicomplexans [72]. The euglenids are part of the Euglenozoa supergroup that includes parasitic kinetoplastids such as *Trypanosoma* and *Leishmania* (Fig 1). Plastids were at one time thought to occur in trypanosomes [73], but this remains unsubstantiated.

## Unique characteristics of endosymbiotic organelles

Mitochondria and plastids are semi-autonomous, containing their own genomes that are inherited independently from the nucleus, often in a non-Mendelian pattern. For both organelles, a uniparental (typically maternal) pattern of inheritance predominates (reviewed in [76,77]). While organellar inheritance of mitochondria is considered strictly maternal in the animal kingdom, this is not true across other kingdoms of Life, where numerous cases of distinct paternal or biparental inheritance are reported for both mitochondria and plastids (reviewed in [78]). Moreover, the dogma of strict maternal mitochondrial inheritance in animals is being overturned by increasingly common reports of paternal leakage in diverse animal species [79–81]. Nevertheless, a preference for uniparental inheritance of mitochondria and plastids during mating is evident throughout Nature, suggesting that possession of a single organellar genotype is advantageous.

It is postulated that uniparental inheritance evolved to minimise the spread of fast-replicating, deleterious, or selfish organellar genome mutations that would create direct competition between divergent intracellular organellar genotypes to the detriment of the host [82–84]. The establishment of endosymbiotic organelles is routinely accompanied by gene transfer of many organellar genes to the nuclear genome of the host, while others are lost entirely, apparently no longer being required by the symbiotic partnership. Nuclear-encoded proteins, both those derived from the endosymbiont genes transferred to the nucleus, and those newly directed from host to endosymbiont, are imported into their respective organelles and are essential for their function, so cooperation and co-adaption between the nuclear and organellar genomes is imperative to the survival of the symbiotic amalgam [85]. Furthermore, uniparental inheritance encourages co-adaption between the 2 genomes and helps to discourage or eliminate genetic conflicts that may arise between them, thus avoiding genetic incompatibility [83,86,87].

## Mechanisms for uniparental inheritance

**Fig 2:**
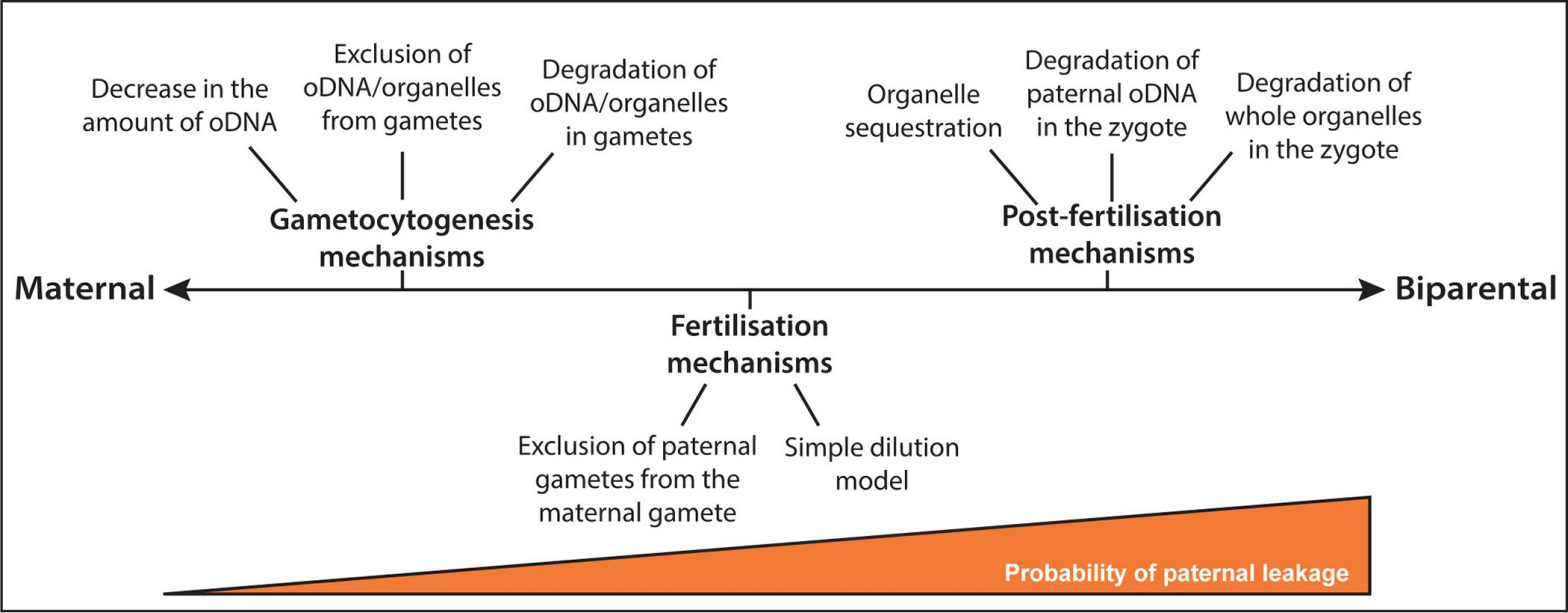
Mechanisms used to achieve uniparental (maternal) inheritance and the probability of paternal leakage or biparental inheritance. This model details the mechanisms commonly used to achieve uniparental (maternal) organellar inheritance of mitochondria and plastids at distinct time points during sexual reproduction, as previously reported (reviewed in [5,76,77,81,88–90]). The orange ramp at the bottom represents our proposal that mechanisms implemented earlier in sexual reproduction should more effectively prevent paternal leakage than those during or after fertilisation. In other words, late mechanisms are potentially more prone to failure resulting in leakage of male derived organelles into sexually produced offspring.

Although uniparental inheritance is widespread, and apparently favourable to the survival of most eukaryotic organisms, there is staggering diversity in the mechanisms that facilitate this uniparental inheritance during sexual reproduction. Intriguingly, variation in mechanisms is observed even in closely related species, suggesting that uniparental inheritance has arisen independently numerous times, perhaps even being lost and regained along multiple evolutionary trajectories. To add to this complexity, there are 3 major stages during sexual reproduction in which uniparental inheritance mechanisms are reported to be implemented: 1/ gametogenesis, 2/ fertilisation, and 3/ post-fertilisation (reviewed in [5,76,77,81,88–90]). It is important to highlight that many organisms use a combination of different mechanisms across these unique checkpoints to increase the chances that paternal organelles are eliminated entirely. Given this, we believe that the timing of these mechanisms directly influences the possibility of a breakdown in maternal inheritance, which is known as paternal leakage. For example, pre-fertilisation mechanisms that degrade or decrease organellar DNA (oDNA) or organelles in gametes prior to fertilisation should be the most effective in deterring paternal leakage because exclusion of organelles from gametes of one sex (canonically males) ensures that they are not delivered to the zygote during mating. In this scenario, any persisting paternal organelles must pass through 2 subsequent phases of sexual reproduction (and their accompanying mechanisms) to become established within the offspring. Mechanisms that occur during later stages of reproduction have fewer opportunities to eliminate one parent’s organelles and, therefore, should be more prone to paternal leakage. Fig 2 details previously reported mechanisms known to facilitate maternal inheritance at each stage of sexual reproduction across the unique kingdoms of Life (reviewed in [5,76,77,81,88–90]) and our view of how efficient we expect them to be at ensuring uniparental inheritance.

## Paternal leakage—Why is it important?

While uniparental inheritance apparently confers some—as yet undefined—fitness benefit, it also carries its own set of evolutionary limitations. The mutation rate of oDNA is frequently higher than in nuclear DNA, likely due to continuous exposure to reactive oxygen species (ROS), limited DNA repair mechanisms, or intrinsic characteristics of organellar ancestry [91–93]. Uniparental inheritance eliminates the opportunity for any kind of purifying selection or recombination between organellar genomes, inevitably leading to an accumulation of deleterious mutations via Muller’s ratchet [76,94]. However, a growing body of evidence suggests that occasional biparental transmission (paternal leakage) may provide opportunities for sporadic recombination to counteract these deleterious effects [83,95–97].

Although uniparental inheritance predominates in sexual crosses within the same species, studies of interspecies hybridisation indicate that control of mitochondrial and plastid inheritance is more tenuous in crosses between different species, and even between individuals from divergent populations of the same species (reviewed in [77,95,98]). This may be due to a breakdown of, or distinct differences in, the mechanisms used to control uniparental inheritance between the 2 individuals and creates the opportunity for recombination of oDNA from a parent that does not usually persist or contribute to the offspring [99,100]. There is direct evidence of paternal leakage of mtDNA after hybridisation in *Drosophila* [101], periodical cicadas [102], mice [103,104], blue mussels [105,106], sea turtles [107], potato cyst nematodes [108], pine trees [109], and maidens’ tears (*Silene vulgaris*) [110]. Similarly, indirect evidence of paternal leakage has been detected through the discovery of recombinant mtDNA genotypes in the great tit [111], conifers [112], silk moths [113], and the cryptococcosis causing fungus *Cryptococcus gatti* [114].

Paternal leakage can also rescue hybrids suffering from cytonuclear incompatibility, where the nuclear and organellar genomes are mismatched and unable to function together [115,116]. Plant hybrids experiencing plastid-specific cytonuclear incompatibilities exhibit traits such as chlorotic leaves and high rates of hybrid mortality. However, such phenotypes were rescued in hybrids where paternal leakage was detected [117–119] because one parental plastid genome is more compatible with the hybrid nuclear background than the other [120–122]. Paternal leakage can rescue cytonuclear incompatibilities by introducing genetic variation among organellar haplotypes and increasing the likelihood that a compatible organellar genome is inherited [76,123]. Interestingly, a similar phenomenon occurs in some parasitic protists that demonstrate transient biparental organellar inheritance followed by segregation of organelles in the offspring (see below [124–126]).

## Mitochondria and plastids as drug targets in parasitic protists

In most, if not all protists, mitochondria and plastids underpin metabolic processes essential for survival. Thus, in parasites such as *Plasmodium* and *Toxoplasma*, the apicoplast and mitochondrion are useful drug targets due to their bacterial-like ancestry and essentiality to the parasite throughout the life cycle [127,128]. Apicomplexan mitochondria possess unique electron transport chain components, metabolic pathways, and protein translation machinery that can be selectively inhibited without interfering with the mitochondria of the host. Likewise, the absence of apicoplasts or plastid-like organelles in human cells makes this compartment a prime target for the development of therapeutics to combat apicomplexan parasites, and several commonly used antimalarial drugs perturb mitochondrial or apicoplast functions to kill parasites [129,130]. However, mutations in organellar genomes can render parasites resistant to some of these drugs (reviewed in [131]), challenging our ability to control parasitic diseases. Thus, it is important to understand how organellar genomes, and the resistance polymorphisms contained within them, are inherited during parasite sexual reproduction and what impact uniparental inheritance has on the spread of resistance through a population of parasites.

## Pathogenic protists with unusual organelles and mysterious sexual cycles

**Fig 3:**
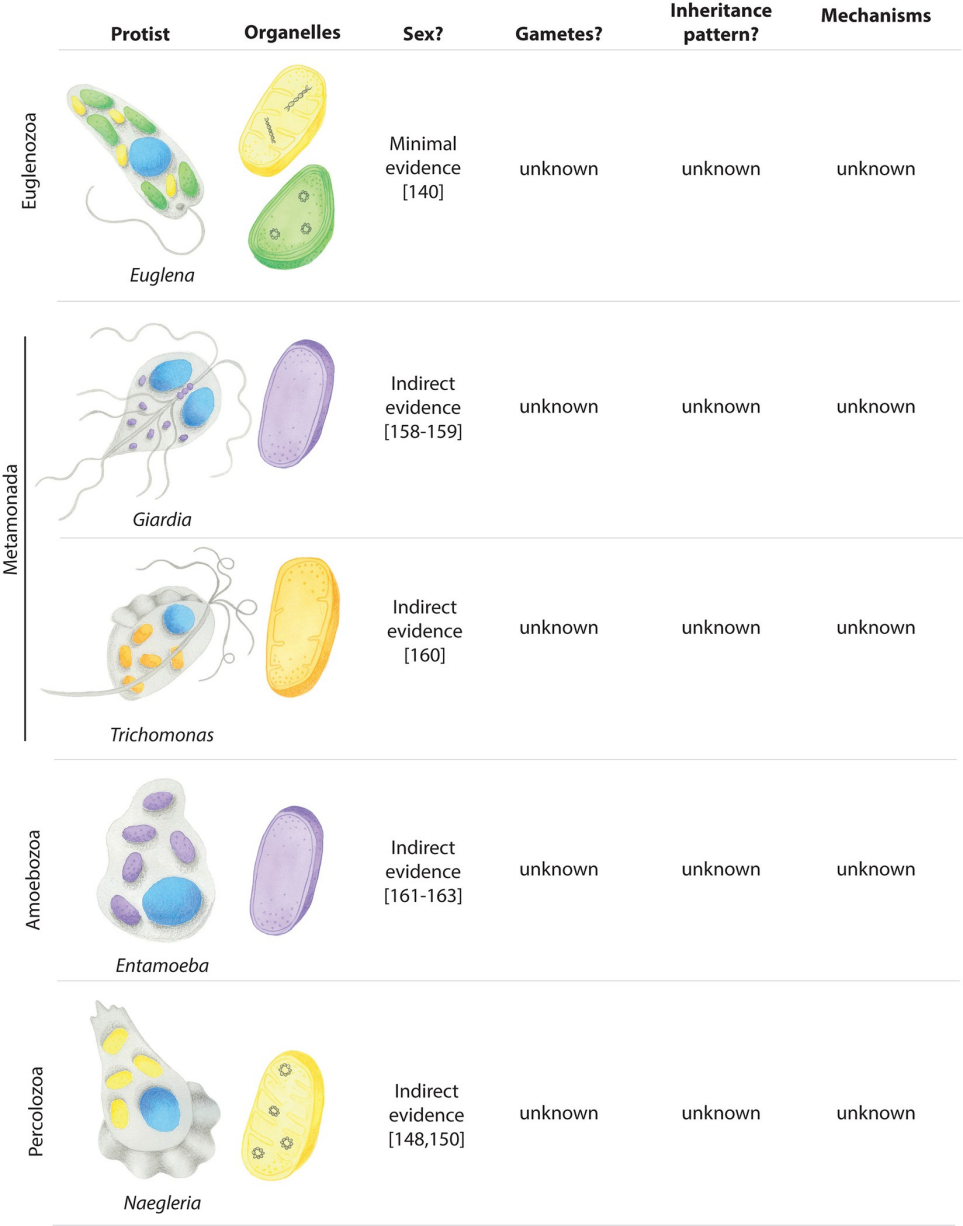
Pathogenic protists with unknown sexual cycles and mitochondrial and plastid inheritance patterns during mating. This figure illustrates the endosymbiotic organelles found in each protist. Protists and organelles are hand-painted by Sarah N. Farrell. Mitochondria (yellow) contain either circular or linear genomes as depicted. Hydrogenosomes (orange) and mitosomes (purple) do not contain an organellar genome. *Euglena* plastids (green) possess a circular genome. References are provided in brackets below the text where relevant.

Understanding mitochondrial and plastid inheritance during sexual reproduction in protist pathogens requires knowledge of the morphology and genomic contents of the endosymbiotic organelles, as well as the protist’s sexual cycle. Our understanding of organellar biology is often more advanced than our understanding of sex in many euglenozoans, metamonads, amoebozoans, and percolozoans (summarised in Fig 3). There are some studies that indirectly describe the sexual cycles of these protists, but they provide little basis for making hypotheses about the inheritance of mitochondria and plastids during mating.

An instructive example is the common photosynthetic euglenozoan *Euglena* that contains around 10 chloroplasts [132,133]. The chloroplast genome of *Euglena gracilis* was among the first ever characterised organellar genomes and a genome sequence followed [50,134]. Alongside these chloroplasts, *Euglena* possesses a massive, reticulated mitochondrion that undergoes extensive morphological modulation throughout the life cycle. During cell division and later stages of development, the mitochondrion fragments into 200 to 500 individual mitochondria per cell [135,136]. These organelles contain distinct mitochondrial genomes, which have been sequenced [137,138]. However, despite extensive study of the endosymbiotic organelles within these protists, evidence of sexual reproduction is confined to a single paper by Biecheler (1937), where she describes sex in a single species of *Euglena* [139], and investigations of organellar inheritance are not available.

*Naegleria fowleri* is a unicellular free-living percolozoan that opportunistically infects the central nervous system of humans, resulting in the fatal disease primary amoebic meningoencephalitis [140]. *N*. *fowleri* exists in aerobic freshwater environments and contains multiple mitochondria during all life stages [141–146]. The mitochondrial genomes of *N*. *fowleri*, and the related *N*. *gruberi*, have been sequenced and suggest both aerobic and anaerobic life-stages within these protists [147,148]. It remains unconfirmed whether *Naegleria* is sexual, but some evidence suggests that these organisms do—or at least did—participate in sexual reproduction. For example, the genome of *N*. *gruberi* encodes homologs of functional meiosis-specific genes [147]. Likewise, the *N*. *gruberi* NEG strain is heterozygotic—a strong indication that these parasites are the product of mating [147]. Moreover, isoenzyme studies suggest that the related, but nonpathogenic, *N*. *lovaniensis* participates in some form of sexual reproduction [149].

Many parasitic protists lack canonical mitochondria but instead contain MROs such as mitosomes and hydrogenosomes [150]. These MROs are typically found in anaerobic protists and have reduced structure and functions compared to canonical mitochondria. In many cases, they lack an organellar genome altogether, with the entire proteome being encoded by the nucleus and imported from the cytoplasm. The metamonad *Giardia intestinalis* is an anaerobic protist containing 40 to 50 mitosomes that lack mtDNA, possess a reduced proteome [55] and function specifically in iron-sulphur cluster biosynthesis [151]. *Entamoeba histolytica* possess a mitosome that also lacks an organellar genome, but, unlike *Giardia*, the *E*. *histolytica* mitosome does not generate iron-sulphur clusters but is instead implicated in sulfate activation pathways [152]. Both giardiasis and amebiasis can be treated using the pyruvate:ferredoxin oxidoreductase inhibitor nitazoxanide, which disrupts anaerobic energy metabolism in the mitosomes of these protists [153,154]. The metamonad *Trichomonas vaginalis* possesses an MRO called the hydrogenosome, which lacks a genome, cristae, and an electron transport chain (ETC) but produces ATP and molecular hydrogen under anaerobic conditions [56,155]. *Trichomonas* causes the sexually transmitted disease trichomoniasis, which can be treated using hydrogenosome targeting compounds such as 5-nitroimidazole and nitazoxanide [153,156]. Again, nothing is known how *Trichomonas* passes down its hydrogenosomes to progeny.

Sexual reproduction has not been directly observed in any of these parasites, but several lines of genetic evidence suggest a current, or recently lost, sexual stage. Population genetics and fluorescence *in situ* hybridisation studies give evidence of sexual outcrossing and genetic recombination in *Giardia*, *Trichomonas*, *Entamoeba*, and *Naegleria* [157–159]. The presence of meiosis-related genes infers a sexual cycle in *Trichomonas*, *Entamoeba*, and *Naegleria* [147,159–161] as do the observed sexual cycles in the close relative of *Entamoeba*, namely, *Dictyostelium* [162]. Furthermore, the existence of retrotransposons in the *Trichomonas* genome [159] and the presence of isozymes in *Naegleria* [149] also provide evidence for a sexual cycle.

While the above evidence suggests that each of these protists does, or at least has the capacity to participate in sexual reproduction, organellar inheritance cannot be studied until their sexual cycles are described. If found to be sexual, many of these protists provide exciting opportunities to study the modes and mechanisms for mitochondrial and plastid inheritance during mating in the context of atypical plastids and mitochondria. For example, it would be particularly intriguing to track the inheritance of MROs such as the mitosome and hydrogenosome to ascertain whether the same rules of mitochondrial and plastid inheritance apply to organelles without a genome, and if the evolutionary pressure to regulate the proliferation of the organellar genome plays a role in the loss of oDNA.

We now turn our focus to protists in which sexual reproduction is documented, and which may provide clues as to the sorts of inheritance patterns and mechanisms we expect to find in these understudied pathogenic protists.

## Pathogenic and nonpathogenic protists with known sexual cycles but unknown patterns of mitochondrial and plastid inheritance

**Fig 4:**
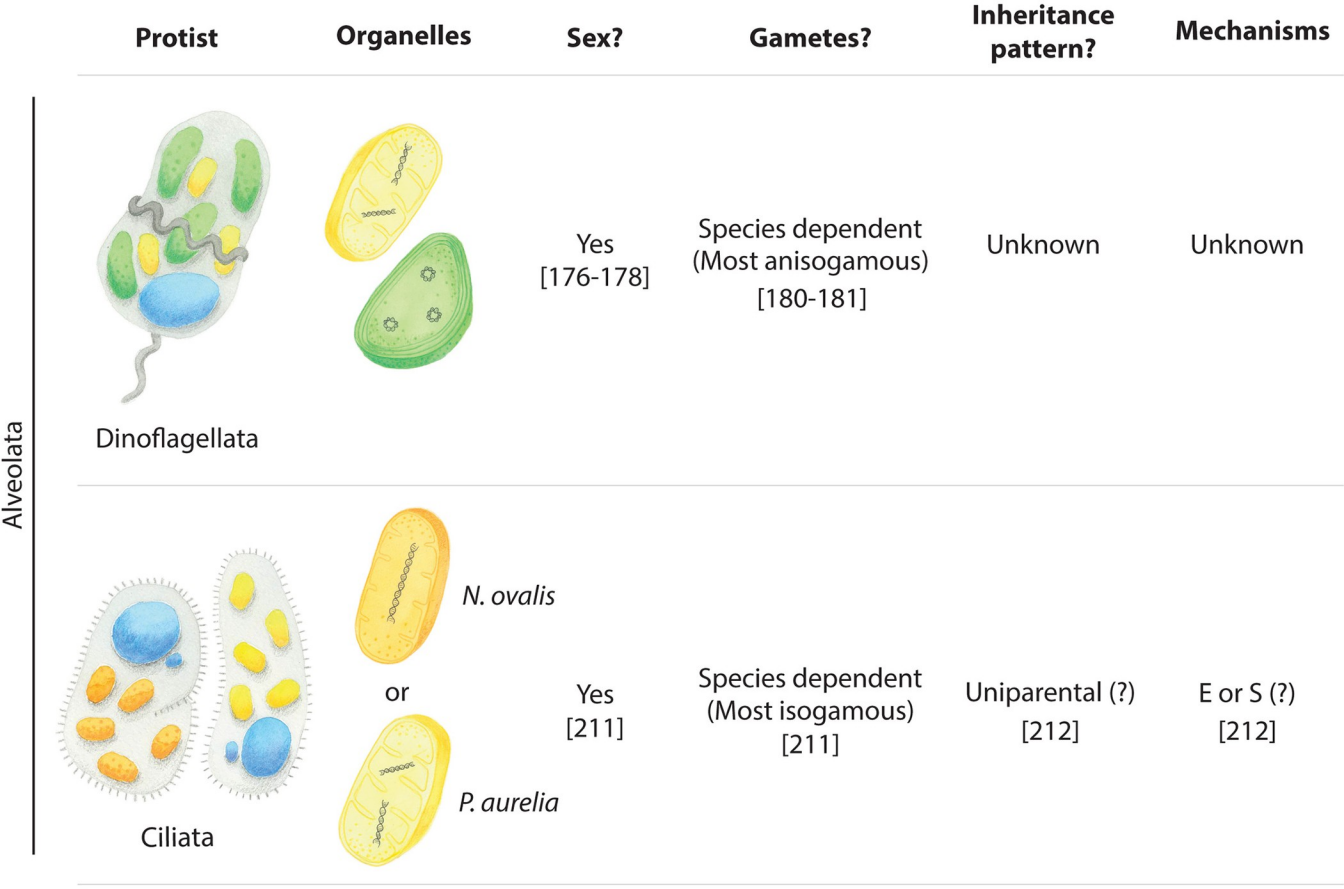
Nonpathogenic protists with known sexual cycles but unstudied mitochondrial and plastid inheritance patterns. Protists and organelles are hand-painted by Sarah N. Farrell. Within the group Ciliata, some ciliates contain hydrogenosomes (orange) with linear genomes (e.g., *N*. *ovalis*) while others possess classical mitochondria (yellow) with linear genomes (e.g., *P*. *aurelia*). Dinoflagellates possess both mitochondria and plastids (green), each harbouring their own genome. Potential mechanisms for uniparental inheritance in these groups include exclusion (E) and sequestration (S). References are provided in brackets below the text where relevant.

In some groups of protists, including dinoflagellates and ciliates, sexual reproduction is well characterised, and we have valuable insights into the presence, morphology, and behaviour of mitochondria and plastids during pre- and post-zygotic stages of their life cycles. Although mitochondrial and plastid inheritance during mating is yet to be directly studied in these protists, we can begin to form hypotheses of possible modes and mechanisms for mitochondrial and plastid inheritance by drawing parallels with related organisms in which these processes are better understood (summarised in Fig 4).

### Dinoflagellates

Dinoflagellates play important and diverse roles in marine and freshwater ecosystems around the globe. Some species are primarily free-living autotrophs or mixotrophs (e.g., *Amphidinium* and *Pyrocystis*), whereas others survive as symbionts, forming mutualistic relationships with marine invertebrates such as corals and anemones (e.g., *Symbiodinium*) [163,164]. Dinoflagellates can also be heterotrophic or parasitic in nature, infecting various organisms including marine crustaceans, molluscs, salps, tunicates, rotifers, annelids, algae, protozoans, and fishes, but not humans [165–170]. Some dinoflagellates are pathogenic to humans, however, producing toxins that accumulate in seafoods and cause ciguatera poisoning or paralytic shellfish poisoning [171].

Dinoflagellate mitochondria are similar to those in animals. There are multiple mitochondria in each cell that contain tubular cristae and discrete genomes [172]. Dinoflagellates plastids are, however, more varied and possess unique features. Most dinoflagellates possess photosynthetic peridinin-containing plastids that are the product of secondary endosymbiosis of a red alga [173]. Intriguingly, the genome of these plastids is uniquely broken into multiple minicircles that are a mere 2 to 3 kb in size and typically encode a single gene each [174]. Some dinoflagellates have discarded their original peridinin-containing plastid for alternative photosynthetic bodies [69,174]. Further, many heterotrophic or mixotrophic dinoflagellates contain plastids that have lost their photosynthetic capabilities [174], and at least 1 dinoflagellate has lost its plastid altogether [71]

Dinoflagellates are capable of sexual reproduction [175–177]. Most reproduction in dinoflagellates is asexual, but certain environmental conditions or stressors induce sexual development [177,178]. Many dinoflagellates produce morphologically similar, or isogamous, gametes, meaning that both mating types are indistinguishable from one another. Others, such as *Noctiluca* and *Pyrophacus steinii*, are anisogamous, meaning they produce morphologically distinct gametes [179,180]. While mitochondria are yet to be observed in dinoflagellate gametes, various studies report the presence of multiple plastids [181]. Interestingly, dinoflagellate gametes are widely reported to possess a reduced number of plastids compared to asexual stage vegetative cells [182–189]. However, ultrastructural studies have revealed the presence of multiple plastids and mitochondria in zygotes and post-zygotic life stages [181,190,191]. Conversely, the number and size of mitochondria appear to decrease as the zygote develops, and plastids are reported to partially degenerate or decrease in number [190,192]. Despite this, multiple mitochondria and plastids have been observed in planomeiocytes (a temporary, motile form that emerges from the hypnozygote shell under favourable conditions) during later stages of sexual development [192]. This is noteworthy because in many other organisms, decrease in the number of mitochondria, plastids, or organellar nucleoids belonging to a particular parental gamete is used as a mechanism for uniparental inheritance. For example, in the Japanese rice fish, *Oryzias latipes*, the number of paternal mtDNA nucleoids is decreased during spermatogenesis to aid in maternal inheritance [193]. Similarly, in the fern *Pteris vittata*, paternal chloroplasts undergo a dramatic 1/15 decrease in volume during spermatogenesis. Subsequently, paternal chloroplasts divide in the absence of chloroplast DNA (cpDNA) replication, further reducing the overall number of cpDNA nucleoids present [79,194]. It is plausible that similar mechanisms may be implemented in dinoflagellate gametes to underpin a uniparental inheritance pattern for these endosymbiotic organelles. That said, studies into the fate of mitochondria and plastids in sexual dinoflagellates, and their inheritance patterns during mating, are yet to be conducted.

Overall, it is plausible that organelles may be inherited from a single parent in dinoflagellates (particular in anisogamous species), and that the degenerating plastids observed in post-zygotic stages may belong to one mating type and be selectively degraded to ensure uniparental inheritance of these organelles. Such phenomena have been observed for mitochondria in *Caenorhabditis elegans* [81,195], mitochondria and plastids in *Volvox carteri* [196], mitochondria in *Dictyoshaeria cavernosa* [197], mitochondria and plastids in several isogamous green algae (reviewed in [79]) and *Chlamydomonas reinhardtii* [198–200]. Understanding the fate of these organelles and their genomes across different dinoflagellate species will offer insight into both the patterns and mechanisms of organellar inheritance and how different lifestyles, organelle origin, and specific function impact the fidelity and mechanisms of uniparental inheritance.

### Ciliates

Ciliates are unicellular protists defined by dimorphic nuclei and the presence of cilia during at least 1 stage of their life cycle. *Balantidium coli* is the only ciliate known to parasitise humans, residing in our gastrointestinal tract. While *B*. *coli* was first described to contain mitochondrion-like bodies lacking cristae or tubules [201], ultrastructural studies of an isolate from the Philippines described the presence of a mitochondrion with cristae [202]. It remains unclear whether this mitochondrion harbours a mitochondrial genome or possesses its own bacterial-like translational machinery. However, the 30S ribosomal subunit inhibitor tetracycline is used to treat balantidiasis, and therefore may function by impeding protein translation in the mitochondrion of this anaerobic ciliate [203].

Unlike their apicomplexan and dinoflagellate relatives, ciliates lack any plastid [204]. However, many ciliates participate in kleptoplasty, where they retain and utilise plastids of an ingested algal prey. The stolen plastids in ciliates appear unable to divide and must be replaced from newly engulfed prey following cell division, so it seems unlikely that they have a dedicated mechanism of inheritance [205,206].

It is well-established that ciliates can occupy both aerobic and anaerobic environments. Aerobic ciliates tend to possess large numbers of classical mitochondria (e.g., *Paramecium*), whereas anaerobic species contain hydrogenosomes, some of which still contain their own mitochondrial genome (e.g., *Nyctotherus ovalis*) [207–209]. Given that there is almost no exchange of cytoplasm or other organelles between 2 conjugants (or gametes) during the unusual ciliate mating [210], mitochondrial inheritance is perceived to be uniparental [211]. As a result, exclusion or sequestration mechanisms appear to be implemented by default in these organisms to achieve uniparental inheritance. Ciliates are amenable to genetic modifications, and in future, ultrastructure or microscopy studies could be applied to test this conclusion or to uncover other mechanisms used to achieve their uniparental inheritance pattern.

## Pathogenic protists with known sexual cycles and traceable patterns of mitochondrial and plastid inheritance

**Fig 5:**
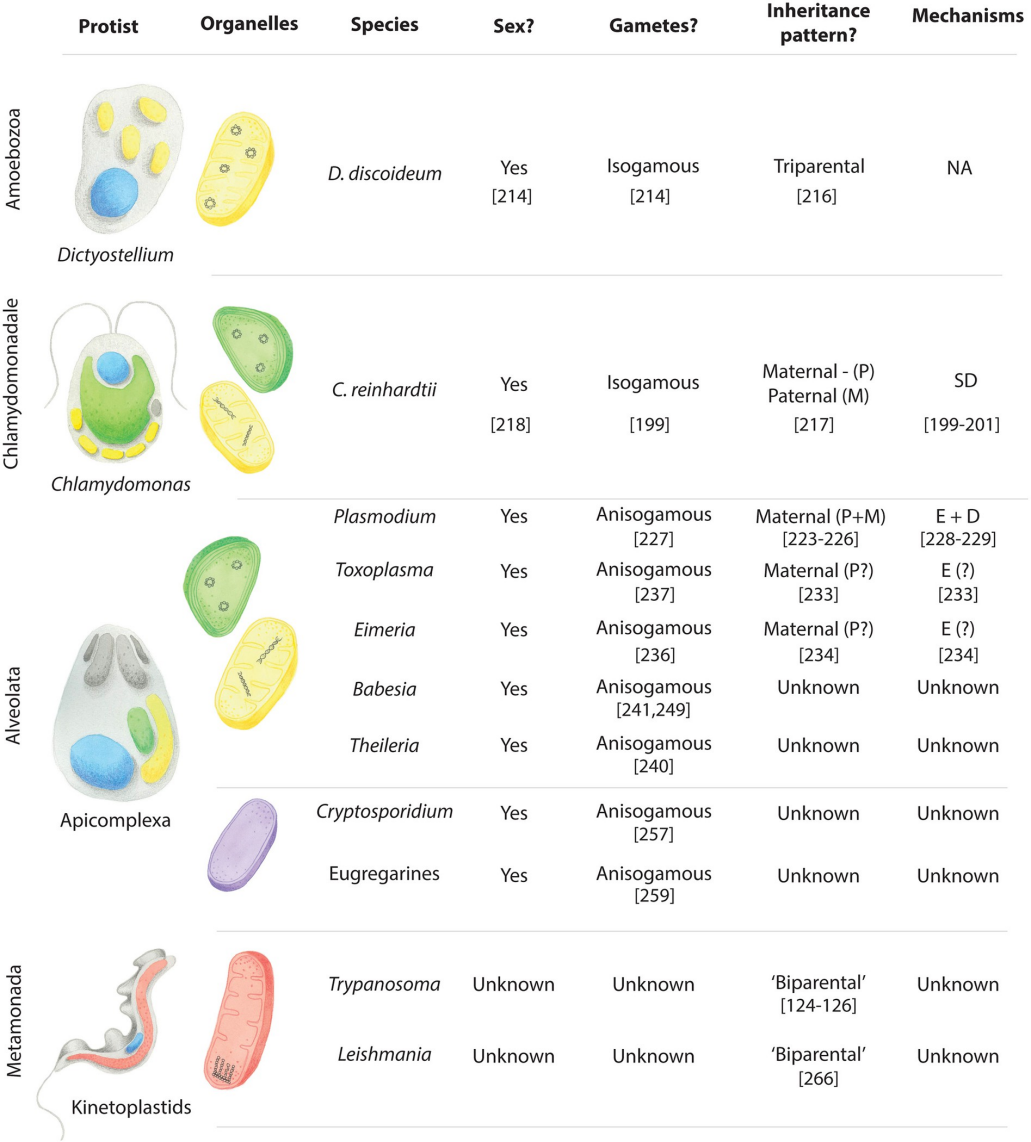
Pathogenic protists with known sexualities and better studied organellar inheritance patterns. This figure illustrates the endosymbiotic organelles and presence or absence of an organellar genome within each listed protist. Protists and organelles are hand-painted by Sarah N. Farrell. Organellar genomes are linear or circular as depicted. While many apicomplexans possess both an apicoplast (green) and a classical mitochondrion (yellow), *Cryptosporidium* and the eugregarines instead contain a single mitosome (purple) and no apicoplast. The mitochondrion found in *Trypanosoma* and *Leishmania* (red) harbours kinetoplast DNA comprised of DNA minicircles and maxicircles. (P) = plastid, (M) = mitochondria. Different mechanisms such as selective degradation of organellar DNA (SD), degradation of whole organelle structures (D) and exclusion (E) are used to ensure uniparental inheritance. References are provided in brackets below the text where relevant.

The few protists where mitochondrial and plastid inheritance during mating has been studied offer some valuable insights into the modes of organellar inheritance and accompanying mechanisms we might expect to find in other related alveolates, metamonads, euglenozoans, and amoebozoans (summarised in Fig 5). Furthermore, the experimental approaches used to study these processes can be applied in other protists to broaden our understanding of mitochondrial and plastid inheritance during mating in these understudied groups.

### *Dictyostelium*: A protist related to the pathogenic *Entamoeba* with a peculiar pattern of mitochondrial inheritance

*Dictyostelium* is a bacterivorous amoebozoan that proliferates as single cells but undergoes significant changes in behaviour and development when starved, forming intimate contacts with surrounding cells of the same strain. During the sexual development that follows, these amoebae differentiate into 3 distinct mating types [212,213]. Gamete fusion in *Dictyostelium* is promiscuous, often leading to the formation of multinucleated syncytia due to a lack of mechanisms to prevent fusion between multiple gametes. Over the next few hours, these syncytia split to form binucleate cells prior to fusion of the nuclei and downstream microcyst development [214]. This results in amalgamation of the cytoplasmic contents of multiple individuals following their fusion. Fusion events between more than 2 gametes frequently leads to triparental mitochondrial inheritance, as the haploid progeny often inherits nuclear DNA from 2 parents alongside the mitochondrial genome of a third [215]. While uniparental inheritance is commonly regarded as the universal and preferred mode of mitochondrial and plastid inheritance during mating in many model organisms, less well-studied organisms such as *Dictyostelium* highlight that alternative modes of inheritance do exist and may be more common than first anticipated.

### *Chlamydomonas*—An example of diverse patterns and mechanisms for uniparental inheritance

The unicellular green algae *Prototheca* (which causes protothecosis in humans and dogs) and the closely related *Helicosporidium* (which parasitises insect guts) possess mitochondria and non-photosynthetic plastids, but nothing is known about inheritance of these endosymbiotic organelles. *Chlamydomonas* is a nonpathogenic, well-characterised relative of these pathogens that may provide clues as to the modes and mechanisms for the inheritance of endosymbiotic organelles during sexual reproduction in these pathogens. *Chlamydomonas reinhardtii* displays interesting patterns of mitochondrial and plastid inheritance in which cpDNA is inherited from the maternal (mt^+^) mating type and mtDNA is inherited from the paternal (mt^-^) mating type [216,217]. Within an hour of zygote formation, mt^-^ cpDNA is selectively degraded by an mt^+^-gamete–specific Ca^2+^-dependent nuclease that is preferentially imported into the mt^-^ chloroplasts [200]. Early work by Sager and colleagues demonstrated that mt^+^ cpDNA was highly methylated, whereas mt^-^ cpDNA was not [218]. Moreover, a DNA methyltransferase CrMET1 is expressed in mt^+^ gametes and targeted to the plastid [200]. Together, this suggests that methylation of mt^+^ cpDNA may protect it from digestion. This is supported by additional studies demonstrating that treatment with methylation inhibitors increases the rate of biparental and paternal inheritance in *C*. *reinhardtii* [219]. However, the link between methylation and maternal inheritance remains controversial, as other studies indicate that selective methylation or hypomethylation of cpDNA does not necessarily correlate with maternal inheritance [220,221]. By contrast, mtDNA from both parents persists in zygotes until the beginning of meiosis in *C*. *reinhardtii*. After meiosis is induced, the maternal mt^+^ mtDNA is selectively degraded, leaving only paternal mt^-^ mtDNA nucleoids [198,199]. The mechanism for selective degradation of mt^+^ mtDNA in the zygote is currently unknown. The difference in timing between the degradation of cpDNA and mtDNA suggests that uniparental inheritance of the respective organelles is controlled by distinct mechanisms in *C*. *reinhardtii* [198].

### The poster child for studying mitochondrial and plastid inheritance in parasitic protists—*Plasmodium*

Mitochondrial and plastid inheritance has been most thoroughly characterised in apicomplexans because their sexual life cycles can be recapitulated experimentally and is often fundamental for disease transmission. All *Plasmodium* species harbour a single mitochondrion and a single non-photosynthetic plastid called the apicoplast. Both organelles contain their own reduced genomes and are believed to follow a uniparental pattern of inheritance [16]. Early genetic cross and DNA blot studies suggested that mitochondria and apicoplasts are maternally inherited in *Plasmodium* [222–224]. Subsequently, maternal inheritance of the mitochondrion has been confirmed in sexual crosses tracking mitochondrion-encoded resistance to the antimalarial drug atovaquone [225]. In *P*. *falciparum*, maternal inheritance of both organelles is likely implemented by exclusion of apicoplasts and mitochondria from male microgametes (sperm). Imaging revealed that both organelles remain lodged within the residual body of male gametocytes during exflagellation (the process in which microgametes/sperm are produced [226]), suggesting that sperm exclusion mechanisms ensure maternal inheritance in *P*. *falciparum* [227]. Intriguingly, Stanway and colleagues reported the absence of the apicoplast in male *P*. *berghei* gametocytes entirely [228]. In this instance, the presence of an additional degradation mechanism suggests that maternal inheritance of the apicoplast may be a more tightly regulated and strict process in *P*. *berghei* compared to other *Plasmodium* species.

In many organisms that employ selective-degradation or exclusion mechanisms to eliminate organelles, the organellar genome is degraded prior to the elimination of the organelle structure. For example, in the green alga *Bryopsis maxima*, preferential destruction of both paternal plastid DNA and mtDNA occurs during late gametogenesis. The remaining plastid structures are eliminated by lysosome-like organelles 24 to 48 h after mating [229,230]. Similarly, the mitochondrial endonuclease G CSP-6 mediates degradation of paternal mtDNA and accelerates breakdown of empty mitochondria in *C*. *elegans* [231]. While such organellar genome reductions are yet to be reported in *Plasmodium*, this may serve as an additional mechanism to ensure maternal inheritance of the apicoplast and mitochondrion in these protists, as depicted in Fig 2.

The absence of apicoplasts and apicoplast-specific markers in male gametes also suggests a maternal inheritance pattern for the apicoplast in *Toxoplasma gondii* and *Eimeria tenella* [232,233]. Interestingly, electron microscopy of *Toxoplasma*—and also *Eimeria*—indicates that mitochondria are present in both male gametocytes and gametes [234]. However, studies of these mitochondrial genomes, and how they are inherited, are yet to be done. In future, it would be intriguing to determine whether the mitochondria packaged into the sperm of these anisogamous [235,236] protists are carried into the zygote, or whether they are excluded from the female gamete during fertilisation as observed in the Chinese hamster *Cricetulus griseus* [237]. Excitingly, recent breakthroughs in inducing sexual development in *T*. *gondii* in mice—thus eliminating the need to harvest sexual forms of the parasite from infected felines—offer a promising and tractable system to study mitochondrion and apicoplast inheritance in this excellent apicomplexan model [238].

*Babesia* and *Theileria* are apicomplexan parasites responsible for livestock and companion animal diseases throughout much of the world [239,240]. Ultrastructural studies of asexual stage parasites indicate that various *Babesia* species possess 1 or 2 mitochondria and a single apicoplast [241,242]. However, the morphology and behaviour of these organelles, particularly during sexual stages of development, is limited to preliminary electron microscopy data for *Babesia* gametocytes and no immunofluorescence, or live cell microscopy studies are available [243]. In *Theileria*, ultrastructural studies have identified the presence of mitochondria in various life stages, and genome sequencing of *T*. *parva* and *T*. *annulate* confirms the presence of the apicoplast in these apicomplexan parasites [35,244]. Surprisingly, the *Theileria* apicoplast is yet to be visualised through electron microscopy or other imaging techniques and is only known through sequencing of the apicoplast genome [245,246]. Overall, very little is known about the inheritance or mechanisms for inheritance of the apicoplast and mitochondria in these organisms, although recent developments in methods to recover *Babesia* gametes in vitro [247] suggests that investigating mitochondrial and apicoplast inheritance in this apicomplexan may soon be possible.

*Cryptosporidium* is the “black sheep” of the apicomplexan family as it has lost the apicoplast and contains a genome-less MRO termed the mitosome [248,249]. Intriguingly, while select gastroinstestine-inhabiting cryptosporidians such as *C*. *muris* and *C*. *andersonii* possess a complete set of tricarboxylic acid (TCA) cycle associated enzymes and a truncated electron transport chain, these are lost entirely from other intestinal-type species such as *C*. *parvum* and *C*. *hominis* [248,250–252]. Ultrastructural analysis of *C*. *muris* revealed a double-membraned mitosome with highly developed cristae like *T*. *gondii* [253]. By contrast, the mitosomes of *C*. *parvum* and *C*. *hominis* are reduced in size and possess atypical cristae [248,254]. Together, this highlights that diversity in mitosome structure and function between different cryptosporidians is most likely based on their unique metabolic requirements and the host environments they occupy. Mitosome inheritance during sexual reproduction is yet to be studied in *Cryptosporidium* species. Sex studies in *Cryptosporidium* have recently become tractable [255], and it will be fascinating to dissect whether the mitosome is uniparentally inherited like the mitochondrion in *Plasmodium*, or if such inheritance patterns become unnecessary in the absence of organellar genomes.

Eugregarines are another group of anisogamous apicomplexans that lack an apicoplast, but these enigmatic parasites are understudied compared to other apicomplexan groups [256,257]. Distinctive mitochondria with tubular cristae have been identified multiple times in gregarines [256,258–262]. Despite this, transcriptomics revealed that mitochondrial metabolism is reduced to differing degrees in many gregarines, with most lacking respiratory complexes III and IV, and some lacking the ETC and TCA cycle entirely [39,40]. As described for *Cryptosporidium*, gregarines present another exciting opportunity to investigate differences in mitochondrial inheritance patterns and/or mechanisms between apicomplexans that do or do not contain a mitochondrial genome and lack an apicoplast.

The overarching theme among apicomplexans is that while most possess an apicoplast and mitochondrion throughout their complex life cycles, patterns of inheritance and mechanisms used to facilitate their inheritance are yet to be properly explored in all but a few tractable models, and some intriguing instances of organelle and/or organelle genome loss pose fascinating scenarios of possible inheritance modes.

### Euglenozoans with peculiar mitochondria and mitochondrial inheritance patterns

The parasitic protists *Trypanosoma* and *Leishmania* contain a single mitochondrion that harbours an unusual network of circular DNA termed the kinetoplast. Kinetoplast DNA (kDNA) is positioned adjacent to the basal body of the single flagellum and comprises an interconnected network of approximately 25 to 50 maxicircles (23 kb in size) and several thousand minicircles (1 kb in size) that together form a disc-like structure [52,53]. Maxicircles encode 20 genes, many of which are cryptogenes whose primary mRNA transcripts require editing to become translatable. Minicircles encode guide RNAs that mediate editing of these maxicircle genes [263]. Interestingly, in *Trypanosoma brucei*, lab-derived hybrid clones contained heterogeneous kDNA networks consisting of minicircles from both parents but maxicircles from a single parent, indicating some form of partial uniparental inheritance [124,125]. It is postulated that the whole kDNA network is initially biparentally inherited but homogeneity of maxicircles is quickly achieved through random DNA segregation during subsequent rounds of mitotic division [124–126]. Conversely, minicircles are not randomly segregated between daughter networks, maintaining the heterogeneity of the originally inherited network [124–126]. Similar patterns of biparental minicircle and maxicircle are also observed in *Leishmania* [264]. In future, it would be intriguing to observe kinetoplast inheritance in these protists during their natural sexual reproduction.

## Final remarks and future directions

Overall, staggeringly little is known about mitochondrial and plastid inheritance during sexual reproduction, or the mechanisms used to achieve this inheritance, in the pathogenic protists discussed in this review. This is largely attributed to our current inability to culture many of these organisms *in vitro* or to easily induce their sexual development in the lab. However, many genetic and molecular biology tools created to study the biogenesis, dynamics, and segregation of mitochondria and plastids in apicomplexan parasites could be applied in other protists to study mitochondrial and plastid inheritance during mating. Fluorescent protein markers have been used extensively in *Plasmodium* and other apicomplexans such as *Toxoplasma gondii* and *Eimeria tenella* to track organelles across the life cycle, and setting up laboratory sexual crosses should now be possible to explore inheritance [247,265–271]. In instances where *in vitro* induction of sexual development or genetic modification is not possible, well-validated live cell mitochondrial stains with different colours such as MitoTracker could be utilised to localise and track the inheritance of mitochondria in gametes, zygotes, and post-zygotic stages.

Ultimately, an understanding of the sex lives of these parasites is fundamental to the study of mitochondrial and plastid inheritance during mating, particularly in the context of reduced organelles such as mitosomes and hydrogenosomes. *Toxoplasma* and *Cryptosporidium* seem ripe for study of organelle inheritance, with the latter presenting an intriguing test case of whether a likely ancestral mechanism of maternal inheritance persists or has been rendered defunct with the loss of the apicoplast and no genome in the mitosome. Protistologists have a lot to learn about the inheritance of these important organelles, so many of which are useful drug targets.
